# Photobiomodulation Induced by 670 nm Light Ameliorates MOG35-55 Induced EAE in Female C57BL/6 Mice: A Role for Remediation of Nitrosative Stress

**DOI:** 10.1371/journal.pone.0067358

**Published:** 2013-06-28

**Authors:** Kamaldeen A. Muili, Sandeep Gopalakrishnan, Janis T. Eells, Jeri-Anne Lyons

**Affiliations:** Department of Biomedical Sciences, College of Health Science, University of Wisconsin-Milwaukee, Milwaukee, Wisconsin, United States of America; University of Pécs Medical School, Hungary

## Abstract

**Background:**

Experimental autoimmune encephalomyelitis (EAE) is the most commonly studied animal model of multiple sclerosis (MS), a chronic autoimmune demyelinating disorder of the central nervous system. Immunomodulatory and immunosuppressive therapies currently approved for the treatment of MS slow disease progression, but do not prevent it. A growing body of evidence suggests additional mechanisms contribute to disease progression. We previously demonstrated the amelioration of myelin oligodendrocyte glycoprotein (MOG)-induced EAE in C57BL/6 mice by 670 nm light-induced photobiomodulation, mediated in part by immune modulation. Numerous other studies demonstrate that near-infrared/far red light is therapeutically active through modulation of nitrosoxidative stress. As nitric oxide has been reported to play diverse roles in EAE/MS, and recent studies suggest that axonal loss and progression of disability in MS is mediated by nitrosoxidative stress, we investigated the effect of 670 nm light treatment on nitrosative stress in MOG-induced EAE.

**Methodology:**

Cell culture experiments demonstrated that 670 nm light-mediated photobiomodulation attenuated antigen-specific nitric oxide production by heterogenous lymphocyte populations isolated from MOG immunized mice. Experiments in the EAE model demonstrated down-regulation of inducible nitric oxide synthase (iNOS) gene expression in the spinal cords of mice with EAE over the course of disease, compared to sham treated animals. Animals receiving 670 nm light treatment also exhibited up-regulation of the Bcl-2 anti-apoptosis gene, an increased Bcl-2:Bax ratio, and reduced apoptosis within the spinal cord of animals over the course of disease. 670 nm light therapy failed to ameliorate MOG-induced EAE in mice deficient in iNOS, confirming a role for remediation of nitrosative stress in the amelioration of MOG-induced EAE by 670 nm mediated photobiomodulation.

**Conclusions:**

These data indicate that 670 nm light therapy protects against nitrosative stress and apoptosis within the central nervous system, contributing to the clinical effect of 670 nm light therapy previously noted in the EAE model.

## Introduction

Experimental autoimmune encephalomyelitis (EAE) is the primary animal model of the human disease, multiple sclerosis (MS), sharing clinical characteristics and pathogenic mechanisms with MS [1]. Both are considered autoimmune, neurodegenerative diseases characterized by chronic demyelination of neurons and eventual loss of axons. Myelin specific CD4^+^ T helper cells are important in disease initiation and progression through the secretion of proinflammatory cytokines, including interferon-gamma (IFNγ), interleukin (IL)-17 and tumor necrosis factor alpha (TNFα) [1]. Conversely, anti-inflammatory cytokines IL-10 and IL-4 have been reported to be important to recovery from EAE and disease amelioration [1].

More recently, oxidative and nitrosative stress have been implicated in EAE/MS pathogenesis, both early and late in the disease process. Qi et al. showed that oxidative injury to mitochondria evident by increased protein nitrosylation preceded infiltration of the central nervous system (CNS) by peripheral immune cells, suggesting that oxidative and nitrosative stress are early events in EAE [2], [3]. In addition, Dutta et al. reported that markers of oxidative stress related to mitochondrial energy depletion are associated with chronic MS, and may explain the irreversible pathology associated with chronic phase of MS [4], [5]. Furthermore, the currently approved therapeutic agents for the treatment of MS target the immune response and slow disease progression but do not prevent disease, providing further evidence that additional mechanisms are at play in disease progression. These data suggest that successful treatment of MS may depend on the development of therapeutic modalities that remediate oxidative stress and preserve mitochondrial function.

Photobiomodulation (PBM) induced by 670 nm light is an alternative therapy shown effective in soft tissue injuries, wound healing [6]–[8] and neurodegenerative diseases [9]–[16]. Mechanistically, PBM is thought to function through intracellular signaling pathways involving NO-mediated mechanisms. These mechanisms are triggered when near infrared (NIR) photons interact with the mitochondrial photoacceptor molecule, cytochrome c oxidase, culminating in improved cellular mitochondrial energy metabolism, increased production of cytoprotective factors and improved cell survival [17]. Deciphering the role of nitro-oxidative stress and neuroprotective strategies in 670 nm NIR-LED photobiomodulation of MOG_35–55_ induced EAE mice are the focus of current research.

Our laboratory previously showed that photobiomodulation induced by 670 nm light reduced clinical severity of EAE with concomitant down-regulation of IFNγ and TNFα and up-regulation of IL-4 and IL-10 [18]. In other models, PBM has been shown to down-regulate nitrosoxidative stress and up-regulate anti-oxidant mechanisms [17], [19]–[21]. As noted above, nitrosoxidative stress was recently suggested to be important in axonal loss associated with disease progression in MS/EAE. Thus, we hypothesized that modulation of nitrosoxidative stress by 670 nm light may offer neuroprotection in the EAE model. Treatment with 670 nm light down-regulated NO production by antigen-primed lymph node cells *in vitro* and iNOS gene expression in EAE mice. These changes were associated with decreased CNS apoptosis in treated mice. These findings suggest that 670 nm NIR-LED photobiomodulation ameliorates EAE by coordinated immune modulation and modulation of nitrosoxidative stress and further support previous data indicating that PBM may be an effective neuroprotective therapy for the treatment of MS.

## Results

### Photobiomodulation by 670 nm Light Attenuates Antigen-specific Nitric Oxide Production in vitro

**Figure 1:**
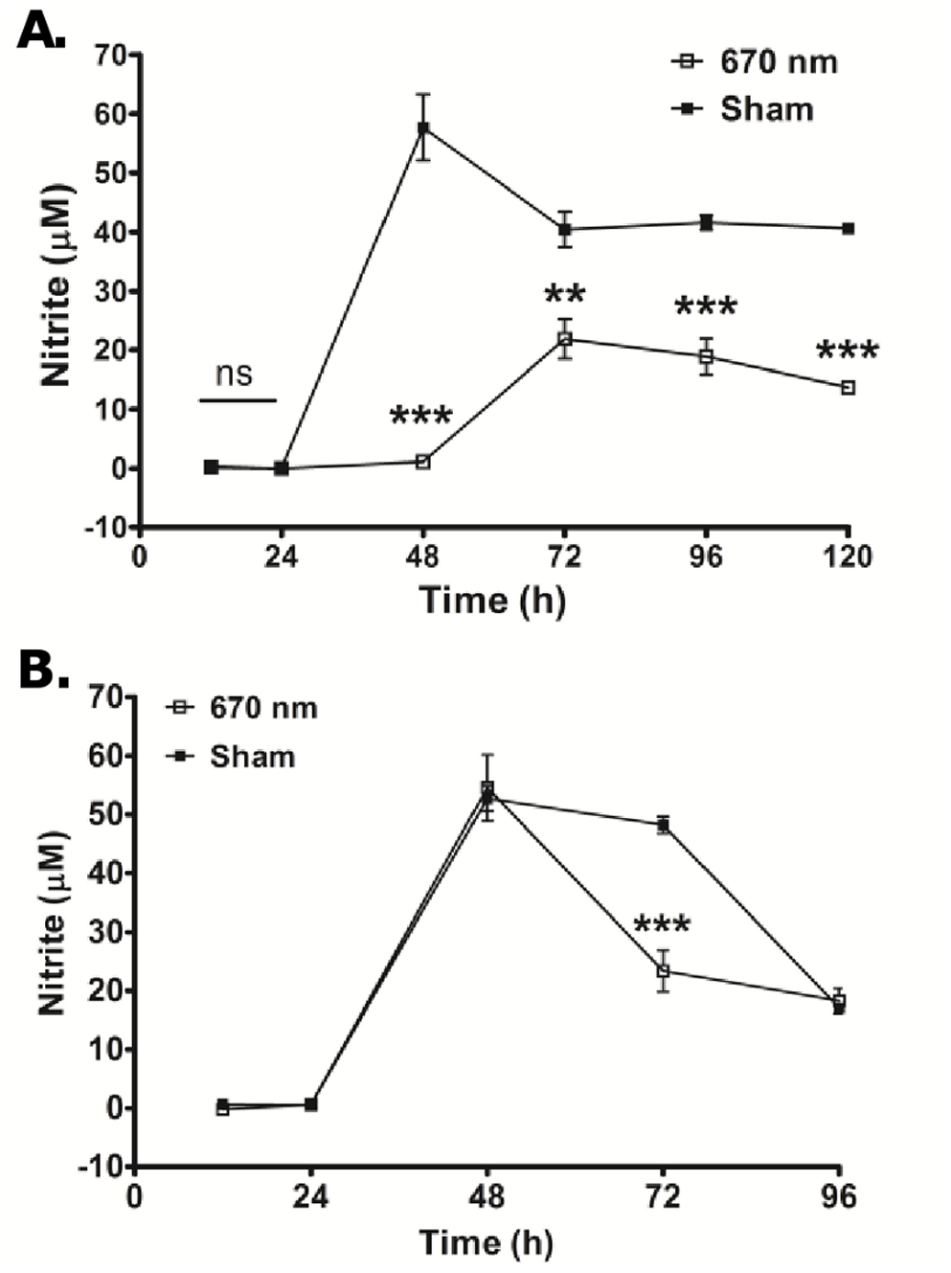
670 nm light modulates nitrosative stress in MOG_35–55_ induced EAE. Pooled heterogenous lymphocytes from peripheral lymph nodes of MOG_35–55_ immunized mice (n = 6–8) cultured *in vitro* with or without (A) MOG_35–55_ or (B) Con A. Cell cultures were exposed to 670 nm (-□-), light or no light (Sham; -▪-) treatment daily for 96 h. Cell culture supernatants were assayed for nitric oxide by Greiss method according to manufacturer’s instructions. **p<0.01, ***p<0.0001 by 2-Way ANOVA. Error bars represents mean ± SD between 3 different experiments.

Our previous data demonstrated amelioration of chronic EAE associated with immune modulation by photobiomodulation induced by 670 nm light [18]. Previous data in other models suggests that photobiomodulation functions in part through down-regulation of nitrosoxidative stress [17], [19]–[21]. To determine if this same effect could be observed in an antigen-specific immune response, we investigated the effect of 670 nm light on antigen-specific NO production *in vitro*. Draining peripheral lymph nodes were isolated from MOG_35–55_-immunized C57BL/6 mice 10 days post immunization (dpi). Single cell suspensions were prepared and cultured with cognate antigen or the mitogenic lectin, concanavalin A (ConA). Cells were treated with 670 nm light or sham control once daily for 96 hours. Nitrite production was measured in cell culture supernatants by the Griess reaction over the course of culture. Data demonstrated that cells treated with 670 nm light produced significantly less nitrite than did sham treated cells (*p*<0.01; Figure 1A) over the entire time-course of the experiment. On the other hand, significant down-regulation of nitrite was only noted at 72 h in ConA treated cells, suggesting that this effect may be antigen specific (Figure 1B).

### 670 nm Light Down-regulates Expression of Inducible Nitric Oxide Synthase (iNOS) mRNA over the Course of EAE

**Figure 2:**
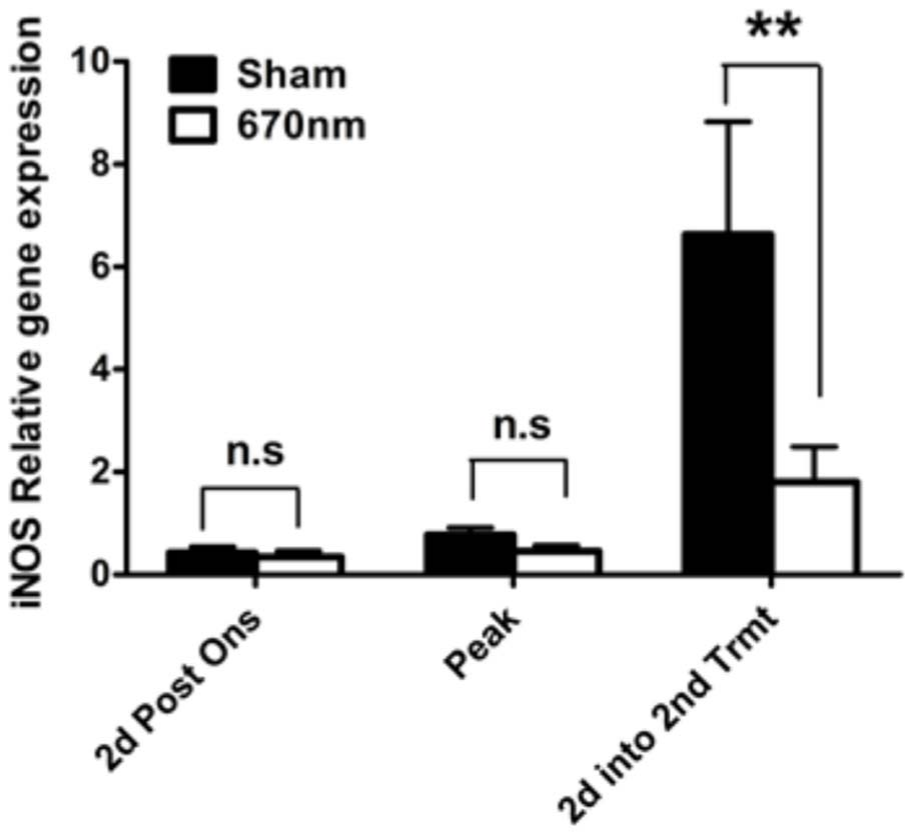
670 nm light reduced spinal cord iNOS mRNA message in MOG_35–55_ induced EAE. WT B6 mice were immunized with MOG_35–55_ for EAE induction and received 670 nm light phototherapy according to the double treatment protocol) or sham (no light treatment). Spinal cord tissues were collected relative to disease course, and cDNA was prepared and analyzed for iNOS gene expression by QPCR. Results were normalized to β-actin and expressed relative to sham control. *, *p*<0.05 by 2-Way ANOVA. Error bars represent mean ± SD between 3 different experiments.

A dual role for nitric oxide in EAE pathogenesis is evident in the literature [22]–[28]. Several studies have demonstrated a contributing role for NO in disease severity and the progression to chronic disease in EAE and MS [4], [5], [29]–[31]. Inducible nitric oxide (iNOS) is the main source of NO in EAE pathology [22], [32]. To determine the effect of 670 nm light treatment on the expression of iNOS over the course of EAE, QPCR analysis was performed on RNA isolated from the spinal cord of mice immunized with MOG_35–55_ and receiving 670 nm light treatment by double treatment protocol (Figure 2)**.** Tissues were collected relative to disease course, as defined in *Materials and Methods*. A significant effect on iNOS gene expression was not observed during the acute episode of disease when mice were treated with the double treatment protocol, but a significant down-regulation was observed later in the disease process, during the chronic phase of disease (Figure 2).

### Importance of iNOS in the Amelioration of Clinical EAE by 670 nm Light

**Figure 3:**
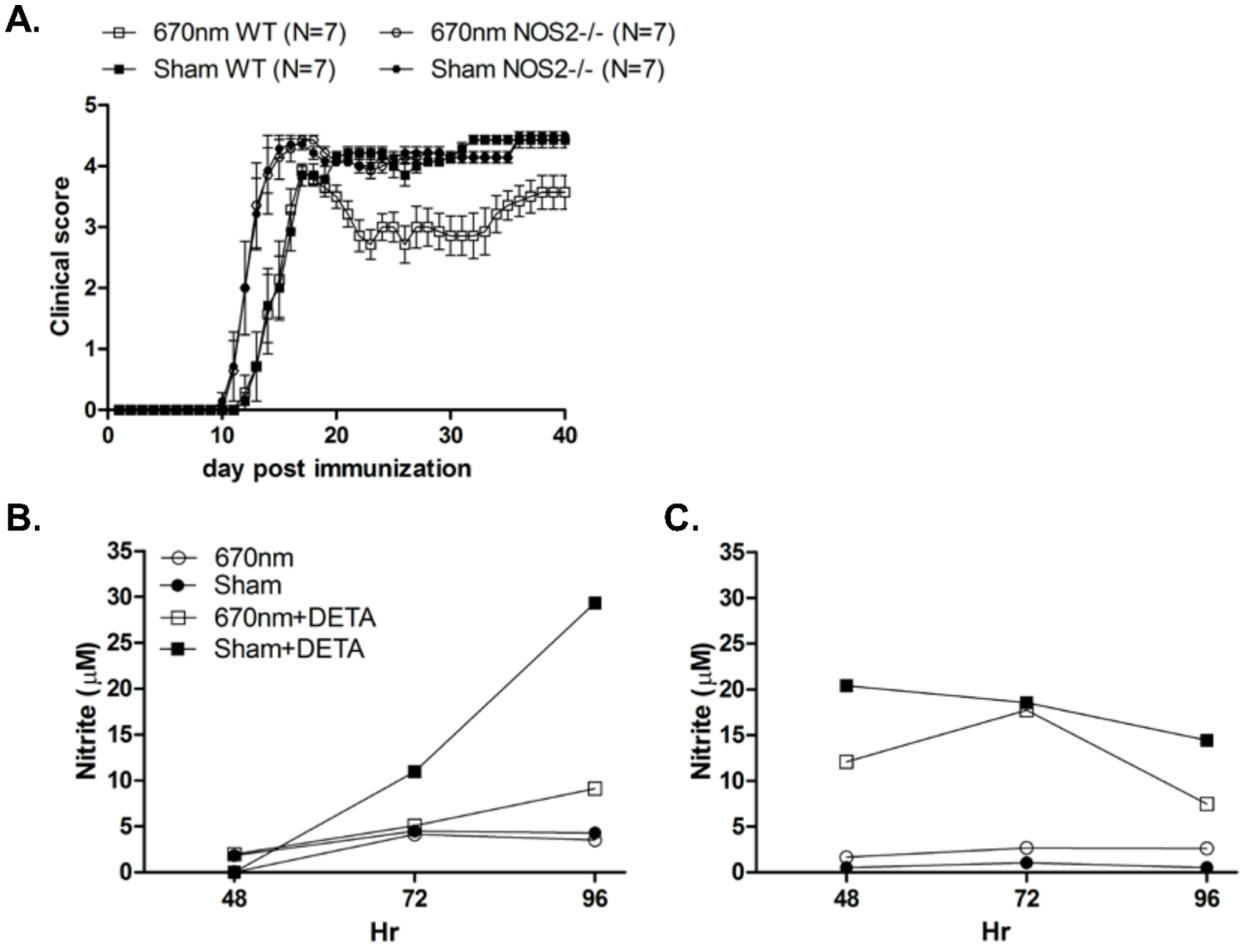
Inducible nitric oxide synthase (iNOS) is important to EAE clinical disease amelioration by 670 nm light treatment. (A) EAE was induced in WT or NOS2^−/−^ B6 mice by immunization with MOG_35–55_ and were treated with 670 nm light phototherapy or sham treatment, according to the double treatment protocol. Clinical disease was assessed over 40 days by a blinded observer; Error bars represent SEM. Data representative of 4 experiments. (B & C) Pooled heterogenous lymphocytes from peripheral lymph nodes of MOG35–55 immunized iNOS−/− mice (n = 4) cultured in vitro were pretreated for 1 hr with or without 100 uM DETANONOate (DETA), prior to 96 h stimulation with (B) MOG35–55 or (C) Con A. Cell cultures were exposed to 670 nm light or no light (Sham) treatment daily for 96 h. Cell culture supernatants were assayed for nitric oxide by Griess method according to manufacturer’s instructions.

Given the current understanding of the role of NO in EAE progression, the down-regulation of iNOS gene expression by 670 nm light observed above would be expected to have a beneficial effect on EAE disease severity. To investigate whether iNOS played a role in the amelioration of EAE severity by 670 nm light, disease was induced by MOG_35–55_ immunization of iNOS knockout (iNOS^−/−^) or wild type (WT) mice, and animals were treated according to the double treatment protocol outlined in Materials & Methods. As previously shown, photobiomodulation induced by 670 nm light resulted in the amelioration of clinical EAE in WT B6 mice (Figure 3). However, treatment with 670 nm light failed to ameliorate EAE in iNOS^−/−^ mice, suggesting that NO produced by iNOS is important to the mechanism of protection by 670 nm light (Figure 3A).

Nitric oxide synthase (NOS), responsible for the production of NO, exists in 3 isoforms. Given the lack of a clinical effect noted in 670 nm-treated iNOS^−/−^ mice noted above, cell culture supernatants from lymph node cells isolated from MOG_35–55_-immunized iNOS^−/−^ mice were subjected to the Griess reaction to measure the production of nitrite (Figure 3B, C). Background detection of NO production was observed in supernatants from 670 nm-treated and sham-treated cells cultured with MOG_35–55_ (Figure 3B) or the mitogenic lectin, ConA (Figure 3C), confirming that iNOS is responsible for the majority of NO production in lymphoid cells [33], [34]. Addition of the nitric oxide donor, DETA NONOate, to cultures resulted in a significant up-regulation of NO production in sham-treated cultures, regardless of whether the stimulation was antigen-specific (Figure 3B) or nonspecific (Figure 3C). Likewise, NO generation was restored in 670 nm, ConA-treated cultures (Figure 3C). However, only a modest up-regulation of NO production was observed in MOG_35–55_-derived supernatants, again suggesting that 670 nm-mediated photobiomodulation may occur selectively with antigen-specific cell activation (Figure 3B).

### 670 nm NIR-LED Light Therapy Results in Reduced Apoptosis in the Central Nervous System

Recent data suggest that the accumulation of oxidative/nitrosative stress in the face of ongoing demyelination contributes to axonal loss and accumulating disability in MS/EAE [2], [5]. Data presented above demonstrate a role for the down-regulation of iNOS/NO in the protective mechanism of 670 nm light in the EAE model. Thus, we characterized the effect of 670 nm mediated photobiomodulation on apoptosis within the spinal cord over the course of MOG_35–55_ induced EAE.

**Figure 4:**
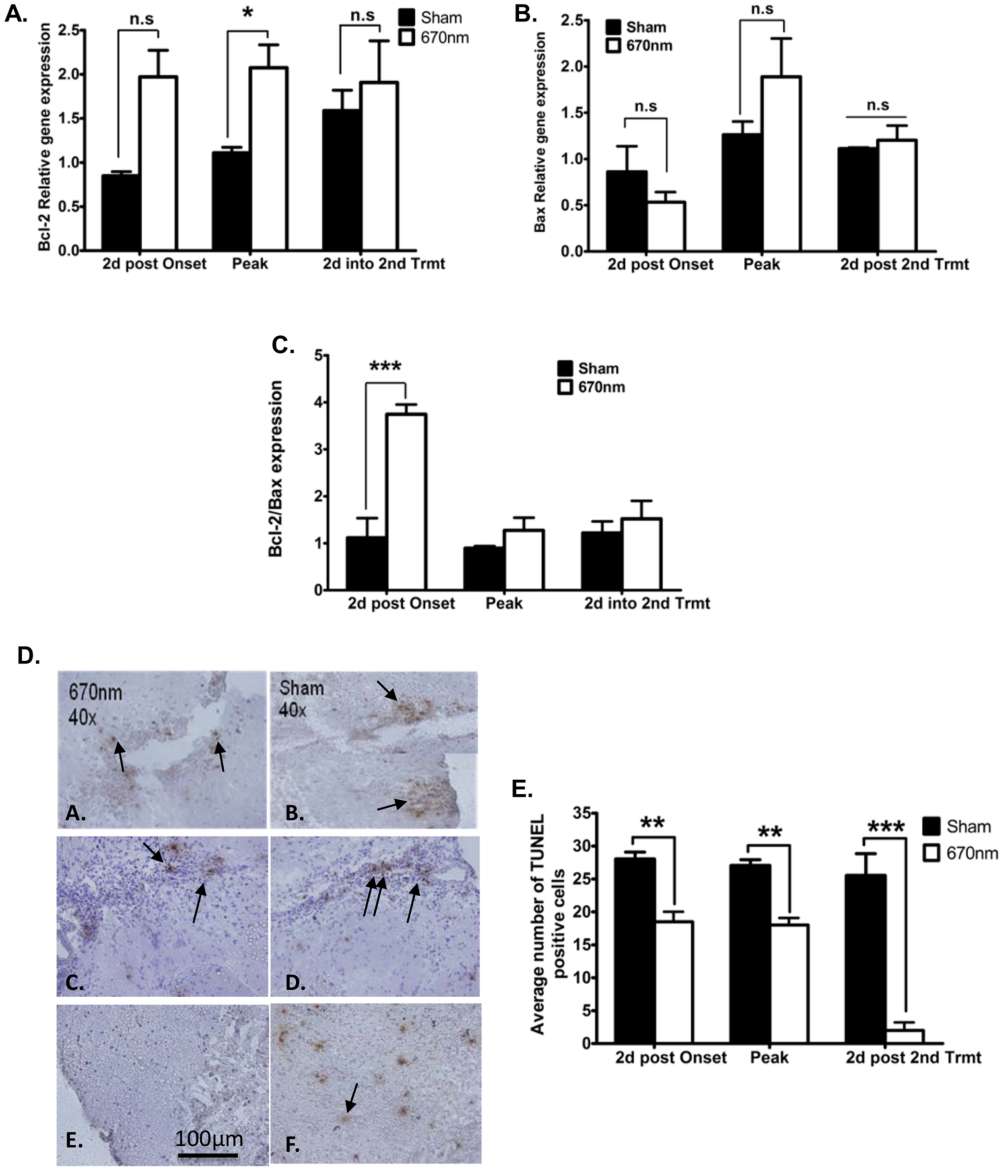
670 nm light regulate apoptosis in EAE mice. EAE was induced in female WT C57BL/6 mice with MOG_35–55_ and animals were treated with 670 nm light therapy for sham treatment according to the double treatment protocol. Spinal cords were isolated from animals at 2 days post first treatment, Peak disease, and 2 days post 2^nd^ treatment and QPCR was performed to assess expression of (A) Bcl-2 and (B) Bax expression, relative to b-actin. (C) Relative expression of Bcl-2 and Bax was calculated and expressed as Bcl-2: bax. (D) For detection of apoposis, 10 µm cryosections were stained using TUNEL assay. Arrows indicate apoptotic cells. Slides are representatives of sections examined from 3 mice per time point. (E) Apoptotic cells from TUNEL stained sections were counted at 40× magnification from 3 fields per section on 3 sections per mouse from 3 mice per treatment group in a single experiment. *p<0.05, ***p<0.01, ***p<0.001, n.s: not significant by 2-way ANOVA.

We first investigated the expression of the anti-apoptotic Bcl-2 and the pro-apoptotic Bax genes important in regulation of programmed cell death. EAE was induced in WT B6 mice by immunization with MOG_35–55_, and mice were subjected to 670 nm light or sham treatment according to the Double Treatment protocol. Quantitative PCR analysis of spinal cord tissue over the course of EAE demonstrated significant up-regulation of the anti-apoptotic gene Bcl-2 at peak EAE in the spinal cord of 670 nm light treated animals (Figure 4A). However, there was not a significant difference in Bax gene expression between 670 nm light treated and sham treated EAE mice at any disease stage tested (Figure 4B). 670 nm light significantly (*p*<0.001) increased Bcl-2 relative to Bax two days after initiation of light treatment (Figure 4C). Together, these data support the hypothesis that 670 nm light therapy may prevent apoptosis in the EAE model.

To directly demonstrate differences in apoptosis over the course of EAE, TUNEL staining was performed on spinal cord sections prepared over the course of EAE from 670 nm light treated and sham treated animals. At all disease stages tested, the number of apoptotic cells observed within the spinal cord of 670 nm light treated mice were significantly lower than that observed in sham treated animals (Figures 4D & 4E). These results, in conjunction with increased Bcl-2 expression relative to Bax, strongly support the hypothesis that down-regulation of iNOS/NO by 670 nm light protects against apoptosis within the CNS over the course of active EAE and would contribute to the amelioration of clinical disease by 670 nm light-mediated photobiomodulation.

## Discussion

EAE is the most studied animal model of human multiple sclerosis (MS), a disease affecting 400,000 Americans, and about 5 million people worldwide. Much of the current understanding of MS pathogenesis and therapeutic advancement in MS management are derived from studies utilizing the EAE model [35]. The current paradigm describes MS as an autoimmune disease mediated by CD4^+^ myelin-reactive T cells that gain access to the CNS and initiate a proinflammatory response leading to the destruction of the myelin sheath by multiple mechanisms [1]. With time, axonal loss accumulates and disease progresses. Most agents for the treatment of MS are effective early in the disease process and act by targeting the autoimmune response [1], resulting in a slower disease progression. However, these agents are ineffective against primary progressive or secondary progressive disease, suggesting non-immune mechanisms are driving disease progression. Previous studies describe nitrosoxidative stress in the CNS over the course of EAE and MS as contributing to disease progression [2], [3], [5]. Published studies demonstrated down-regulation of nitrosoxidative stress by near infrared/far-red light induced photobiomodulation [19], [20], [36], [37]. We previously reported amelioration of clinical EAE associated with immunomodulation induced by 670 nm light in MOG_35–55_ induced EAE in C57BL/6 mice [18]. In this report, we demonstrate that the amelioration of clinical EAE by 670 nm light therapy is also associated with the down-regulation of nitrosoxidative stress and reduced CNS apoptosis in the MOG_35–55_-induced model of EAE.

The present studies utilized the Double Treatment Protocol, in which animals are treated for two separate 7 day periods, separated by a 7 day rest during which no light is administered to the animals [18]. This protocol was developed after early experiments demonstrated a temporary treatment effect by 670 nm light when mice were treated with a single 7 day or 10 day treatment protocol [18]. Also noted in previous studies was a worsening of clinical signs in treated animals if treatment periods were extended past 10 days (unpublished data). The biphasic dose-response curve is well-established [38]. However, the worsening of clinical signs with increasing dose observed in the EAE model has not been seen in other model systems and could be related to the autoimmune pathology of MS and dual roles of nitric oxide on the immune response and disease pathogenesis. Thus, the Double Treatment Protocol results in a sustained clinical effect by 670 nm phototherapy while minimizing potential adverse side effects associated with over-treatment.

In this study, *in vitro* treatment of heterogeneous lymph node populations isolated from MOG_35–55_ immunized mice with 670 nm light demonstrated decreased NO generation compared to sham treated cultures in culture supernatants by the Griess reaction. Similar to the effect of *in vitro* treatment of lymph node cells on the generation of proinflammatory cytokines [18], this effect appeared to be antigen specific, given that similar down-regulation of nitric oxide following 670 nm light treatment was not seen with nonspecific activation with a mitogenic lectin. Thus, it is possible that photobiomodulation induced by 670 nm light could be effective in treatment of antigen-specific autoimmunity, like multiple sclerosis.

The effect of 670 nm light therapy on iNOS gene expression over the course of EAE differed with disease course when administered by the double treatment protocol. In these experiments, no effect on gene expression was noted during the acute episode, while a significant down-regulation was observed during relapse. As demonstrated by Qi et al, evidence of mitochondrial dysfunction and NO generation was evident within the CNS prior to immune infiltration and disease onset [2]. Treatment initiated at disease onset would not inhibit early generation of NO, but would hinder the generation of NO with subsequent relapse. Alternatively, but not mutually exclusive, a significant down-regulation iNOS/NO with relapse would support the role for nitrosoxidative stress in disease progression during the chronic phase, as demonstrated by the work of Dutta et al [4], [5]. Further studies characterizing the mechanism of iNOS/NO down-regulation and the role of NO in disease pathogenesis will be necessary to elucidate these mechanisms.

Previous reports in other model systems demonstrated induction of NO production by NIR light [39], [40], whereas the current work demonstrated down-regulation of NO in the EAE model when utilizing the double-treatment protocol. A comparison of the literature reveals a number of parameters which could contribute to the differential effects of phototherapy on NO production, including treatment parameters, experimental conditions, and the cell populations studied. Treatment parameters, including wavelength and fluence, affect the outcomes of phototherapy [39]–[42]. Those systems that demonstrated up-regulation of NO utilized 780 nm light [39] or broad-band (400–800 nm) visible light [40], whereas our studies, as well as those of Song et al, showing down-regulation of NO utilized 670 nm (current study) or 632.8 nm [41]. Sharma et al. demonstrated down-regulation of inflammatory mediators, including NO, at low fluence but up-regulation of these same mediators at high fluence [42]. Experimental parameters, such as the timing of the assays could also affect the observed results. Other systems characterize the generation of NO immediately following, or shortly after, exposure to NIR light. Our studies focus on NO generation in the days and weeks following NIR light exposure. In addition, the down-regulation of NO observed in the CNS microenvironment could be affected by the complex interaction between the immune response and the CNS microenvironment. This hypothesis is supported by preliminary data from our lab showing a brief up-regulation of iNOS gene expression by lymph node cells within 2 hours of 670 nm light exposure. Furthermore, we noted an initial up-regulation of iNOS gene expression during the preclinical stage of disease when 670 nm light treatment was initiated the day after immunization and continued through the onset of disease. With this same treatment protocol, iNOS gene expression was down-regulated during the acute phase of disease. The observation of divergent effects of NIR light on NO production indicates that careful optimization of treatment parameters will be necessary for phototherapy to be clinically useful. In addition, care in moving forward with the application of phototherapy for the treatment of MS is also prudent: given the relapsing-remitting nature of early disease, it is possible that exacerbation of clinical signs and symptoms may be noted if patients are treated in the period prior to onset of a relapse.

The observed effects of 670 nm light on NO production and iNOS gene expression would be expected to have a beneficial effect on EAE/MS disease progression. Thus, mice lacking iNOS expression (iNOS^−/−^) were immunized with MOG_35–55_ for initiation of EAE and received 670 nm light therapy according to the double treatment protocol. Although disease severity was ameliorated in WT mice, we failed to observe a similar effect in the iNOS^−/−^ mice, demonstrating that the clinical effect of 670 nm light therapy in the EAE model is dependent on iNOS gene expression. This may seem counter-intuitive given that 670 nm light down-regulates iNOS expression and NO generation. However, this could suggest a role for, or compensation by, other forms of NOS, including neuronal (n)NOS or endothelial (e)NOS in EAE pathogenesis or the clinical effect of 670 nm light. Alternatively, this observation could be due to the potential multiple roles of NO in the regulation of EAE/MS pathogenesis, as discussed above.

Down-regulation of oxidative stress protects against MS/EAE disease progression through protection from apoptosis [3], [43] [44], [45]. Indeed, decreased apoptosis within the CNS was noted in animals receiving 670 nm light treatment. While the phenotype of the cells undergoing apoptosis in untreated animals was not determined, cell localization and physical characteristics suggest that early in the disease process (acute disease), these were primarily infiltrating immune cells. With disease progression, apoptotic cells appeared to be resident glial cells.

Apoptosis in the CNS has been linked with a number of factors including cytokines and reactive radicals [46]. Our previous data [18] as well as data presented here suggest that photobiomodulation by 670 nm light therapy could be acting via both mechanisms–down-regulation of proinflammatory cytokines and NO–to protect against disease progression. Protection could be mediated via independent mechanisms and/or through coordinated actions. Free radicals are known to be involved in cytokine signaling [47], [48]. Conversely, cytokine expression, particularly INFγ, affects NO generation [49], [50]. Previous work demonstrated an effect of 810 nm light, also within the NIR/FR region of the spectrum and postulated to act through interaction with cytochrome c oxidase, on the activation of the transcription factor, NFκB [51]. NFκB is known to play a role in the regulation of cytokine signaling and iNOS expression [49], [52]. Interestingly, Espejo et al reported the modulation of oxidative stress by IFNγ, particularly by CNS glial cells [53], further contributing to the potential mechanism of protection afforded by 670 nm light on the clinical course of EAE. These intriguing ideas are under further investigation.

In the current study, we demonstrate down-regulation of nitrosative stress and protection against apoptosis within the CNS by 670 nm light mediated photobiomodulation in the EAE model of MS. These findings, together with our previously published report [18] demonstrate that clinical effects of 670 nm light therapy are in the EAE model, and perhaps other applications of light therapy, are due to multiple and complex mechanisms. Our studies document the potential therapeutic value of NIR/FR light therapy for the treatment of MS and suggest that this noninvasive therapy may provide the neuroprotection necessary to have a lasting effect on disease pathogenesis in the MS patient.

## Materials and Methods

### Ethics Statement

Animal studies were approved by the UWM Animal Care and Use Committee prior to initiation. Animals were housed in and AAALAC accredited facility in accordance with University and NIH guidelines.

### Animals

Specific pathogen-free female C57BL/6 (B6) wild type (WT) and iNOS^−/−^ mice on the B6 background were bred in-house from breeding pairs purchased from Jackson Laboratories (Bar Harbor, ME). Mice were maintained in micro-isolator cages in an AAALAC-accredited facility in accordance with University and NIH guidelines. All animals were supplied with food and water *ad libitum* and maintained on a 12 hour light/dark schedule in a temperature and humidity-controlled environment.

### Antigens and EAE Induction

The mouse MOG_35–55_ peptide (MEVGWYRSPFSRVVHLYRNGK) was synthesized (GenScript, Piscataway, NJ) and purity confirmed by HPLC. EAE was induced in mature mice (6–8 weeks old) by immunization with 100 µg MOG_35–55_ peptide emulsified (1∶1) in IFA with 300 µg *Mycobacterium tuberculosis*, strain H37RA (Difco Labs, Lawrence, KS). Each mouse received 0.05 ml emulsion (s.c.) at four sites and pertussis toxin (300 ng i.p.; List Laboratories, Campbell, CA) at 0 h and 72 h post immunization. Animals were followed for the development of EAE and graded clinically on a scale of 0–5 by a blinded observer (0: healthy, no signs of EAE; 1: loss of tail tone; 2: hind limb weakness/wobbly gate; 3: paresis or paralysis of one hind limb; 4: paralysis of both hind limbs; 5: dead or moribund).

### LED Treatment

Gallium/Aluminum/Arsenide (GaAlA) LED arrays (75 cm^2^) of 670 nm (LED bandwidth 25–30 nm at Full Width Max Power [FWHM]) were obtained from Quantum Devices (Barneveld, WI). Unanesthetized mice were placed individually into a polypropylene restraint device (12.7×9×7.6 cm), and the LED array was positioned directly over the animal at a distance of 2 cm, covering the entire chamber and exposing the entire dorsal surface. Treatment consisted of once daily irradiation at 670 nm for 3 min, at a power intensity of 28 mW/cm^2^ (total power output: 2100 mW) and an energy density of 5 J/cm^2^ (375 J daily total). As indicated, “restraint only” stress was employed, in which mice were placed in the restraint device for 3 min but not exposed to light. Mice were treated with the Double Treatment Protocol, consisting of once daily treatment for 7 consecutive days starting at EAE onset (clinical score  = 1.0), resulting in a total of 3024 J at the level of the spinal cord administered to the animal over the course of treatment. This was followed by 7 day rest period, and a subsequent 7 d treatment period (6048 J total energy administered). Clinical disease was followed for an additional 7 d following cessation of the second treatment period [18].

### RNA Isolation and Quantitative Polymerase Chain Reaction

Mice were anesthetized with a ketamine cocktail and perfused with 60 ml cold PBS via cardiac puncture. Total RNA was isolated from the spinal cord (SC) and peripheral lymph nodes (PLN) tissues using the Trizol method according to manufacturer’s instructions (Invitrogen, Carlsbad, CA). RNA was further purified utilizing the RNEasy kit utilizing genomic DNA eliminator columns and subsequent on-column DNase treatment to eliminate genomic DNA contamination (Qiagen, Valencia, CA). RNA (2 µg) was reverse transcribed using the Transcriptor First Strand cDNA Synthesis Kit (Roche Applied Science, Indianapolis, IN) according to the manufacturer’s instructions. Probe-based quantitative real-time PCR was performed for inducible nitric oxide synthase (iNOS), Bcl-2, and Bax. β-actin was included as the housekeeping gene. Primer/Probe sets were designed using the Universal Probe Design software (Roche). Primers were purchased from Sigma (St. Louis, MO), and probes were purchased from Roche. Amplifications were performed using TaqMan® Universal PCR Master Mix (Roche) on a SmartCycler (Cepheid, Sunnyvale, CA), programmed for 95°C for 10 min, followed by 40 cycles of 95°C for 15 s and 60°C for 1 min, with detection of signal during the annealing/amplification stage. Results were calculated via the Pfaffl method (Pfaffl 2001) and are expressed as fold change in 670 nm treated mice relative to gene expression in samples isolated from sham treated animals at the same disease stage.

### Nitrite Determination

Draining PLN were pooled from 4 mice 10 days post immunization (dpi) with 100 µg MOG_35–55_. Single cell suspensions were prepared, and cells (2.5×10^6^/mL) were cultured in supplemented RPMI 1640 [10% FCS, penicillin (100 U/mL)/streptomycin (100 µg/mL), L-glutamate (2 mM), Sodium pyruvate (0.1 mM), 2-mecarptoethanol (50 mM)] in 96-well flat-bottom plates (BD Biosciences, San Jose, California, USA) in the presence or absence of 10 µg/mL MOG_35–55_ peptide. Stimulation of cells with concanavalin A (ConA; 1 µg/mL) served as a positive control for cell viability. For experiments with cells isolated from iNOS^−/−^ mice, 100 µmol/L of the NO donor [diethylenetriamine (DETA)-NONOate (DETA); Sigma, St. Loius, MO] prepared in sterile PBS was added to cell suspensions, as indicated. Cells were spiked with donor at 48 hours to maintain 100 µM/L of chemicals in the cells, based on the half-life of these chemicals. Cell cultures were treated once daily with 670 nm light or no light treatment beginning 2 h after plating and continuing at 24 h intervals for 96 h. Cell culture supernatants were isolated at 48 h, 72 h, and 96 h, and analyzed for nitric oxide generation was determined by measuring the accumulated nitrite in cell culture supernatants using the Griess reaction (Promega, Madison, WI). In brief, an equal volume of Griess reagent (1% sulfanilamide, 0.1% naphthalene diamine HCI, 2% H_3_PO_4_), was added to cell culture supernatant. Following 10 min incubation at room temperature, color production was measured at 550 nm on a Synergy™ HT Multi-Detection Microplate Reader (BioTek, Winooski, VT), and the nitrite level was calculated according to a standard curve of sodium nitrite (NaNO_2_) in concentrations of 0.15 to 10 µmol/ml.

### Detection of Apoptosis by Terminal Deoxynucleotidyl Transferase (TdT)-mediated dUTP Nick End Labeling (TUNEL)

Apoptosis was detected using TUNEL method according to the manufacturer’s instructions (GenScript, Piscataway, NJ). 5–10 µm cryosections of OCT (Sakura, Tissue-Tek) embedded SC tissues were air-dried at room temperature for 20 minutes, and fixed in freshly prepared 4% paraformaldehyde in 1× PBS for 20 minutes. Sections were rinsed in fresh PBS and endogenous peroxidase activity quenched for 10 minutes at room temperature with 0.3% H_2_O_2_ in methanol. Sections were then permeabilized for 5 min on ice with a solution of 0.1% Triton X-100 and 0.1% sodium citrate. Labeling was done using TUNEL reaction mix (45 µl Equilibration Buffer, 1 µl biotin-11-dUTP and 4 µl TdT), incubated in a moist tray at 37°C for 60 minutes in the dark. Label was removed and sections were incubated with strepavidin-HRP at 37°C for 30 minutes in a moist chamber. Fragmented DNA was detected using DAB substrate solution (5 µl of DAB buffer, 1 µl of 30% H_2_O_2_ in 94 µl of fresh PBS). Slides were rinsed and counterstained with methylene blue, mounted with glycerol and examined with light microscope. Dark brown nuclei representative of apoptotic cells were counted by a blinded observer. Three high-power fields were counted per section, 3 sections per slide, 2 slides per mouse, and 2–3 mice per group. Apoptotic cells were counted in areas of pathology. Results are expressed as average apoptotic cells. Controls include positive and negative sections treated with DNase 1 prior to labeling or absence of TdT from the TUNEL reaction mix respectively.

### Statistical Analysis

Data were analyzed and statistical analyses were performed using GraphPad Prism 5.0 (GraphPad, La Jolla, CA USA). Clinical course was compared by 2-way ANOVA. Recovery analysis was compared by 1-way ANOVA. Multiple comparisons performed by Dunn’s Multiple Comparisons test. Mann-Whitney U-test and Student’s t-test performed, as appropriate. *p*<0.05 considered significant.
